# Clinical outcomes and radiologic assessment of a modified suture button arthroscopic Latarjet procedure

**DOI:** 10.1186/s12891-019-2544-x

**Published:** 2019-04-16

**Authors:** Jian Xu, Haifeng Liu, Wei Lu, Weimin Zhu, Liangquan Peng, Kan Ouyang, Hao Li, Daping Wang

**Affiliations:** Department of Sports Medicine, Shenzhen Second People’s Hospital, Shenzhen First Affiliated Hospital, Shenzhen University, No.3002 Sungang West Road, Futian district, Shenzhen, 518000 Guangdong Province China

**Keywords:** Arthroscopic latarjet, Graft, Fixation, Clinical outcome, Radiologic assessment, Suture button, Modification, Retrospective study

## Abstract

**Background:**

As several neurologic and hardware complications have been reported with screw fixation. Suture buttons are used to serve as an alternative to screw fixation to obtain better outcome and to reduce the complication. The purpose of this study was to observe the clinical outcomes and make the radiologic assessment of a modified suture button (MSB) arthroscopic Latarjet procedure.

**Methods:**

A total of ninty-one patients with recurrent shoulder joint dislocation who underwent MSB arthroscopic Latarjet procedure was retrospectively reviewed. Fifty cases identified from the chart review met the inclusion criteria. The clinical outcomes and position of the grafts, glenohumeral degeneration, and graft healing condition were assessed postoperatively in a follow-up with at least one and half of a year.

**Results:**

All the fifty patients were satisfied with their clinical outcome. The overall complication rate was 4% in this study. The mean visual analog scale score, the affected shoulder active mobility in Ers(external rotation at the side), Era(external rotation in abduction) decreased significantly; the ASES score, Rowe score, Walch-Duplay score improved significantly. CT scans in the sagittal view showed that grafts in 88% of cases were in good position, grafts in 12% of cases were fixed too superiorly and inferiorly. In the axial view grafts in forty cases were flush with the glenoid rim, ten were considered as too lateral. The ten grafts became remodeled and were more flush with the glenoid rim in the follow-up.

**Conclusions:**

The MSB arthroscopic Latarjet procedure provides excellent outcome with few complications, and no degenerative changes were observed in the follow-up. Moreover, the graft fixed too laterally presented a phenomenon of remodeling and became flush with the glenoid rim over time.

**Electronic supplementary material:**

The online version of this article (10.1186/s12891-019-2544-x) contains supplementary material, which is available to authorized users.

## Background

The arthroscopic Latarjet procedure, which involves transferring the coracoid bone with its attached conjoint tendon to the glenoid using arthroscopic technique, has been shown as a reliable and safe treatment option for recurrent anterior shoulder instability [1–6]. However, the arthroscopic technique is relatively complicated with a long learning curve, which leads to complications due to insufficient experiences [7, 8]. Complications including glenohumeral arthritis, graft osteolysis, prominent hardware or screws, graft malpositioning, and graft non-union, are significantly related to fixation methods [3, 9]. Presently, the common fixation method uses two metallic screws. If the length of the metallic screws is too long, irritation of the rotator cuff may occur [2, 10]. Meanwhile, too short metallic screws may cause loosening of the graft or non-union [11].

It seems critical to make sure whether impingement due to screws occurs on the humeral head or not. Moreover, degenerative changes are not only related to the hardware but more related to a non-anatomic fixation of the graft too lateral to the glenoid. Moreover, if the grafts are not flush with the glenoid after fixation using two metallic screws or the screws were oblique to the plane of the glenoid with some of the heads above the glenoid, impingement of the humeral head may happen. Especially when the grafts’ position was too lateral in relation to the glenoid, can lead to degeneration of shoulder joint [12, 13]. As several neurologic and hardware complications have been classically reported with screw fixation, Boileau et al. [14] presented a novel fixation method using suture buttons, which was proven to be an alternative to screw fixation to obtain an excellent outcome. We believed that this fixation is similar to suture button fixation for distal tibiofibular syndesmosis which is an elastic fixation allowing minimal motion [15]. This method might help relieve or reduce impingement of the humeral head caused by the hardware or the bone graft above the glenoid level. On the basis of his study, we modified the technique by lessening the number of portals using only three portals, preparing coracoid openly with a small incision, adding an anti-rotation knotless anchor for coracoid block fixation.

The purpose of this study was to assess whether the MSB arthroscopic Latarjet procedure can reduce over time or avoid impingement of the humeral head and finally achieve a better outcome. We hypothesized that the coracoid graft above the glenoid plane can become remodeled and can be flush with the glenoid without causing degenerative glenohumeral changes after using our MSB Latarjet procedure.

## Methods

Final approval of an exemption from review by an institutional review board was obtained for this study because it was retrospective in nature. When ordering the preoperative and postoperative low-dose 3D CT scans, we have routinely discussed the risks and benefits of 3D CT with our patients. Informed consent was received from all the patients.

### Patient selection

A total of 91 patients who were diagnosed with recurrent shoulder joint dislocation combined with glenoid defect between October 2013 and September 2016 were reviewed. The inclusion criteria were as follows: 1) patients who obtained their shoulder recurrent anterior dislocation with an instability severity index score(ISIS)of more than 6 [16]; 2) patients who received arthroscopic Latarjet procedures using double suture buttons for fixation. The exclusion criteria were as follows: 1) patients who received arthroscopic Latarjet procedures using double metallic screws for fixation; 2)those who received rotator cuff or avulsion fracture repair previously; 3)those who had no or minimal glenoid deficiency and those with isolated labral or isolated Hill-Sachs lesions; 4) those who were followed up less than 24 months.

### Surgical technique

**Fig. 1:**
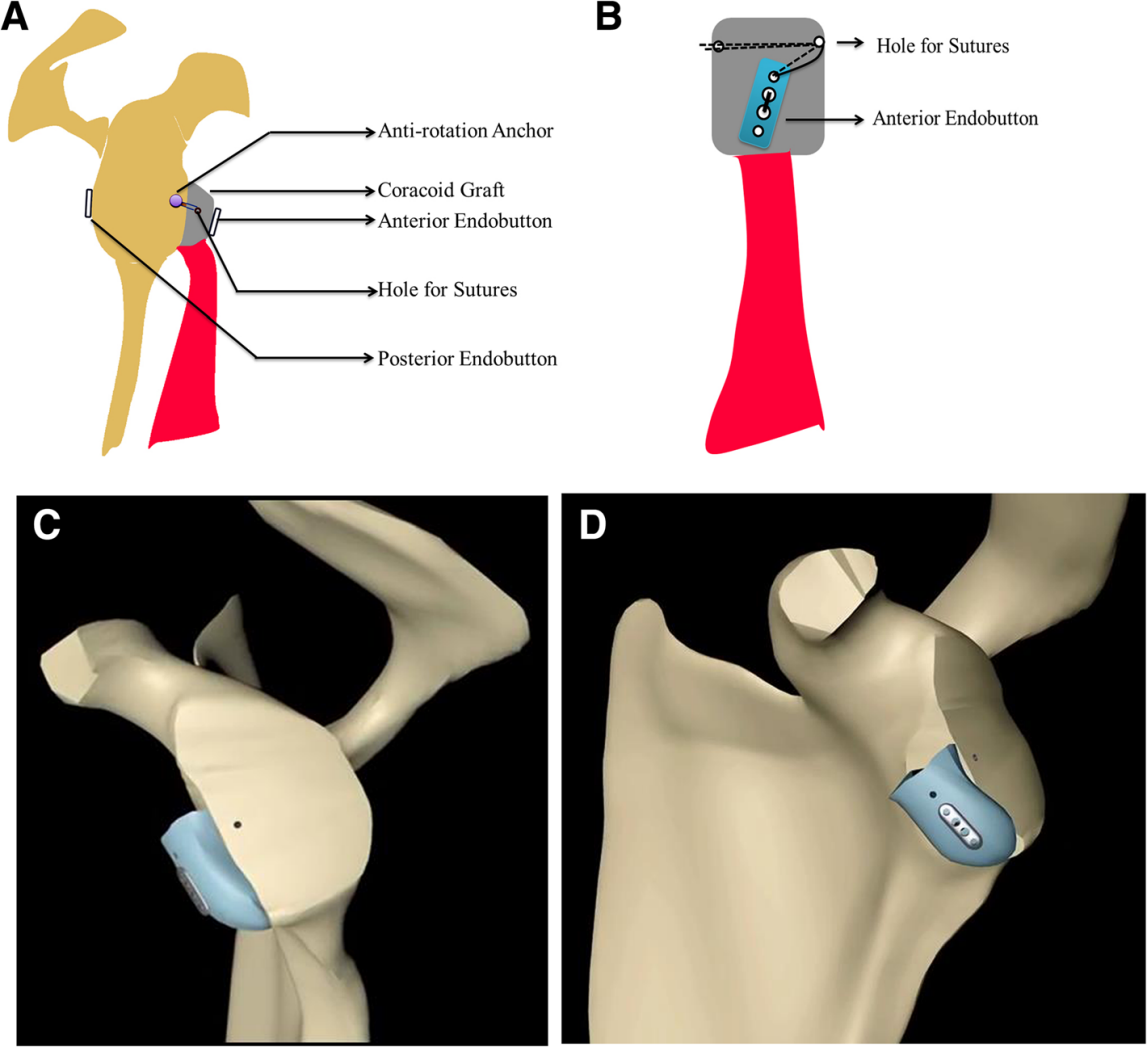
Schematic diagram for the MSB Latarjet procedure. **a-b** 2D view: (**a**):Diagram in the sagittal view. **b** Frontal view of the graft. **c-d** 3D view: C: En-face view. **d** Lateral view

The procedure was performed under general anesthesia associated with an interscalenic block with the patient in the beach chair position. The surgical technique included one step performed openly and other two steps arthroscopically (Fig. 1) (Additional file 2).

#### Step 1: coracoid preparation, drilling, and osteotomy

**Fig. 2:**
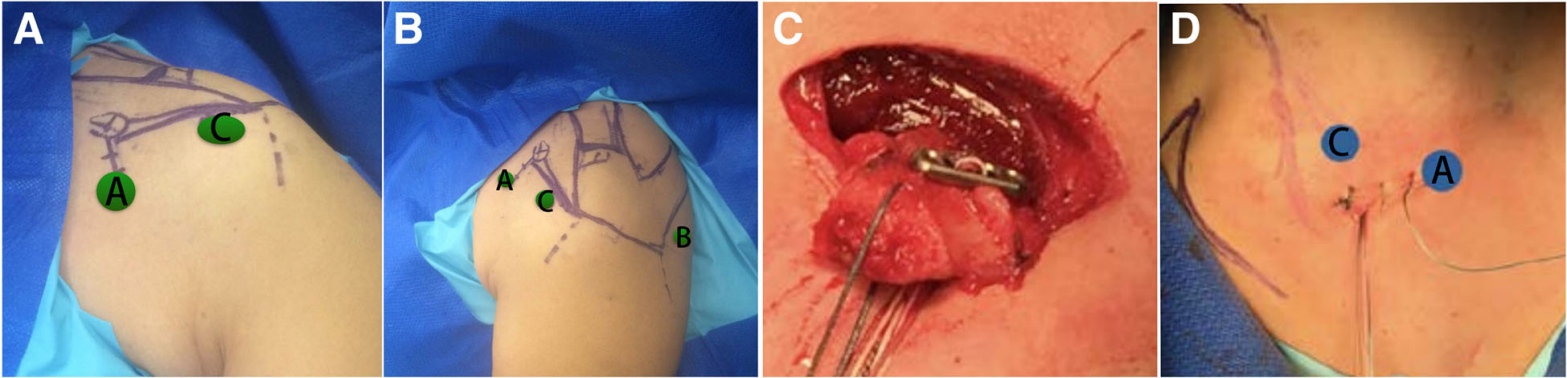
Surgical portals and incision. **a** Front view: The anterosuperior portal (A with black color) which is left by the partly closed incision on the coracoid with 5 mm left. An anterosuperior lateral portal (C with black color) was also established through the guidance of arthroscopy. **b** Lateral view: A standardized posterior portal (B with black color) was established. **c** An incision measuring 2.5 cm was made, which began from 1 cm under the coracoid process in the direction of the axilla. An osteotomy of the coracoid process was performed, and two bone tunnels were drilled with a distance of 6 mm in the cut bone block along its axis. High-strength sutures were pulled into the central hole of a suture button and then pulled together to the proximal bone tunnel. **d** After freshening the bone graft, the incision was partly closed with 5 mm left as the anterosuperior portal

The first step was performed openly. An incision measuring 2.5 cm was made, which began from 1 cm under the coracoid process in the direction of the axilla. The coracoacromial ligament and part of the pectoralis minor muscle were first cut 1 cm from the border of the bone. With the help of an oscillating saw, an osteotomy of the coracoid process was performed at its bend, so that it measured approximately 20 mm long. Two bone tunnels were drilled with a distance of 6 mm in the cut bone block along its axis. High-strength sutures were pulled into the distal tunnel. Three high-strength sutures were pulled into the central hole of a suture button and then pulled together to the proximal bone tunnel (Fig. 2). After freshening the bone graft, the incision was partly closed with 5 mm left as the anterosuperior portal (Fig. 2), which was exactly in the anterosuperior side of the subscapular tendon (Additional file 1).

#### Step 2: glenoid preparation and subscapular muscle splitting

**Fig. 3:**
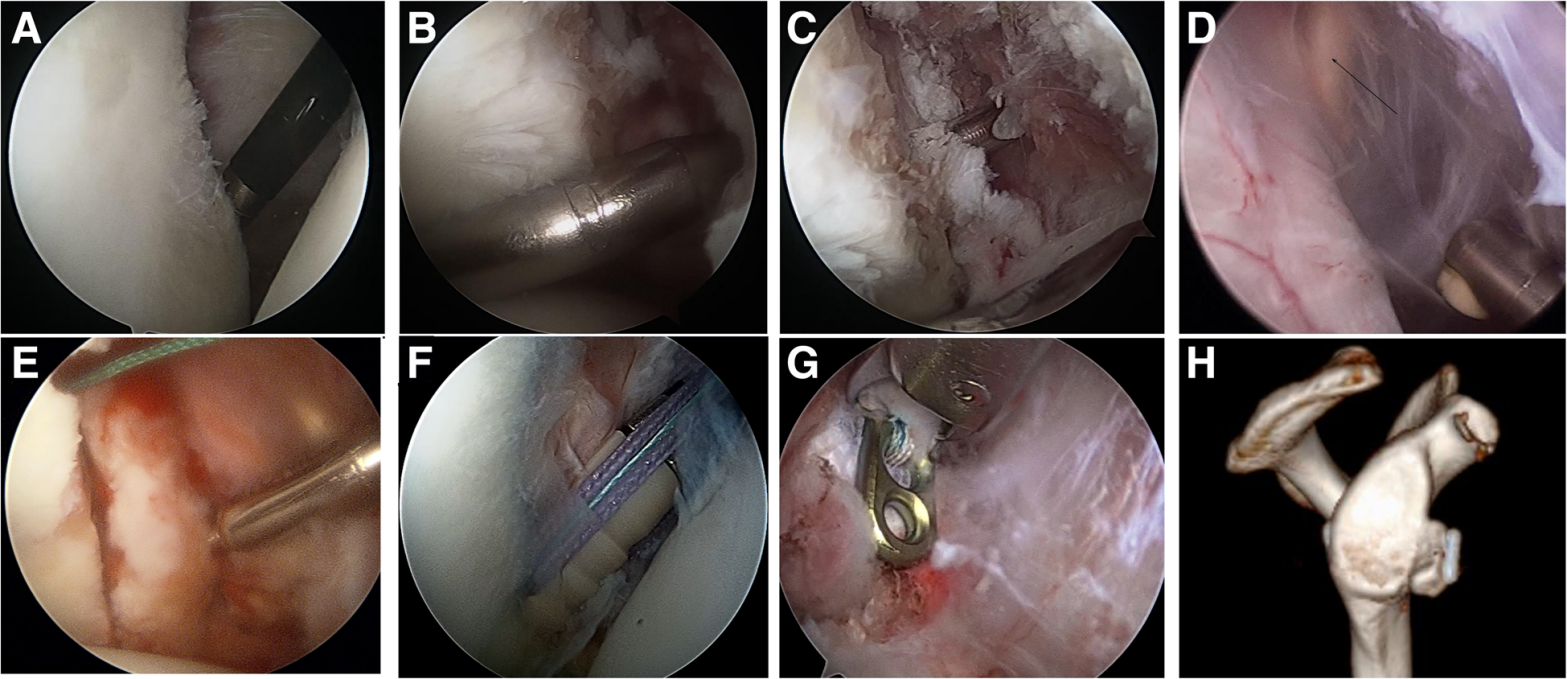
The surgical steps for MSB Latarjet procedure. **a** The glenoid bone defect was exposed. A marker was made on the anterior glenoid edge at half past 3 o’clock for location by the radiofrequency. **b** Debridement was performed on the glenoid and the switching stick was used to locate the accurate position for en-face graft fixation. **c** A tunnel with a diameter of 4.5 mm was drilled into the glenoid at the pre-prepared location with the guidance of custom-made guiding instrument. **d** The axillary nerve was exposed in case of injury. **e** High-strength sutures in the proximal tunnel of the bone block were passed into the tunnel of the glenoid. Then the coracoid block was pulled into the shoulder joint using the three sutures and firmly adhered to the glenoid. **f** The other suture in the distal tunnel was fixed together with a knotless anchor to the glenoid at half past 3 o’clock to prevent coracoid block rotation. **g** The suture button’s position was adjusted to prevent itself from the impingement of the humeral head. **h** The en-face view of the graft position from the CT scan at postoperative day 1

**Fig. 4:**
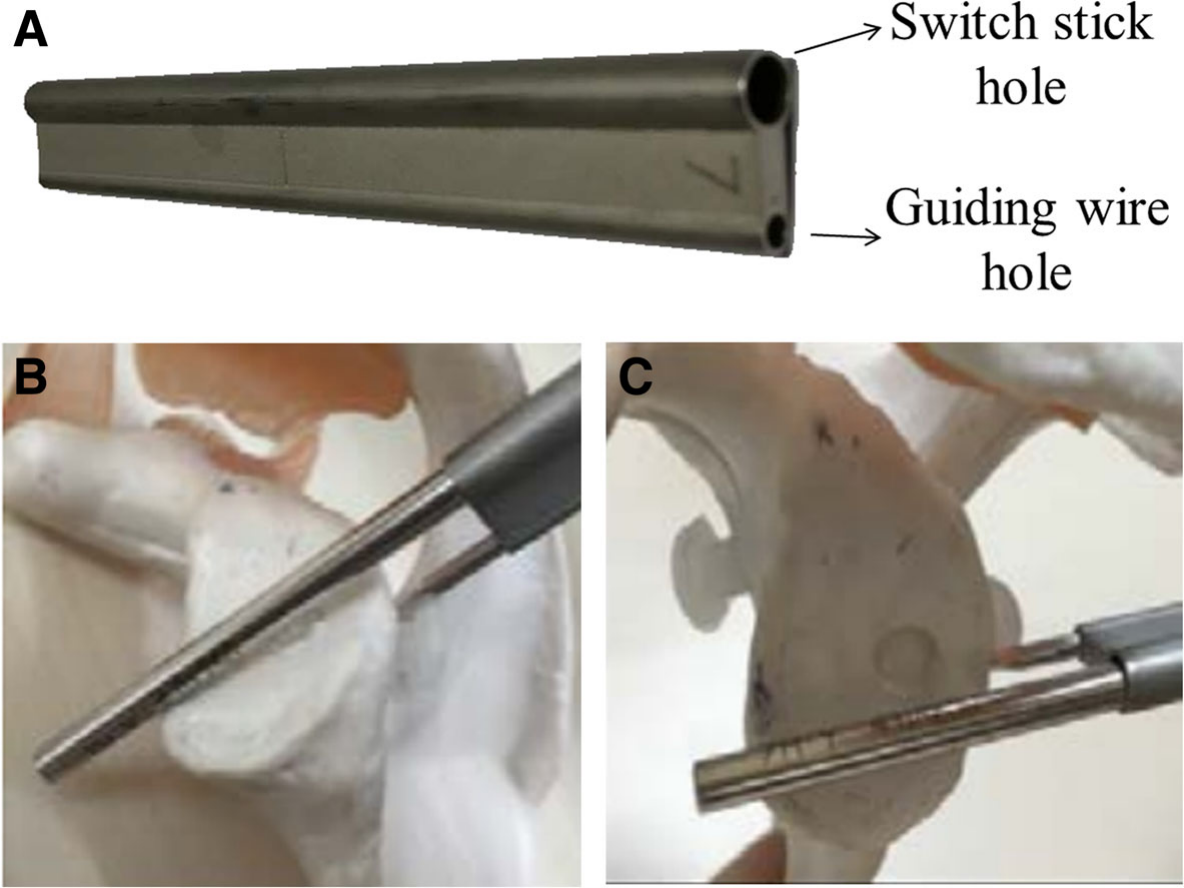
The custom-made guiding instrument for the guiding wire drilling and sutures passing. **a** The instrument has two holes, one for switching stick and one for guiding wire. **b-c** lateral and en-face view while it was put to the glenoid model

The second step was subscapular muscle splitting and glenoid graft bed preparation. A standardized posterior portal (Fig. 2) was established, and another anterosuperior lateral portal (Fig. 2) was also established through the guidance of arthroscopy (Fig. 2). The glenoid bone defect and Hill–Sachs injury could be observed (Fig. 3). A marker was made on the anterior glenoid edge at half past 3 o’clock for location by the radiofrequency. Afterward, a tunnel with a diameter of 4.5 mm was drilled into the glenoid at the pre-prepared location with the guidance of custom-made guiding instrument (Figs. 3 and 4). Subsequently, the axillary nerve was exposed in case of injury (Fig. 3). The subscapularis was split with the help of a switching stick, mainly the muscular portion (Additional file 1).

#### Step 3: coracoid block transferring and fixation

The third step included coracoid block transfer and fixation. Three high-strength sutures in the proximal tunnel of the bone block were passed into the tunnel of the glenoid. The coracoid block was pulled into the shoulder joint using the three sutures and firmly adhered to the glenoid (Fig. 3). The other suture in the distal tunnel was fixed together with a knotless anchor to the glenoid at half past 3 o’clock to prevent coracoid block rotation (Fig. 3). Finally, the relationship between the glenoid and bone block was observed. When the coracoid graft was fixed too laterally under arthroscopic view, some debridement was made to flush the en-face further (Additional file 1).

### Postoperative rehabilitation plan

The patient’s arm was immobilized in a sling in internal rotation for analgesia for six weeks. Rehabilitation protocols were standardized. Pendulum exercises were performed several times per day, beginning on postoperative day 1. No active exercises or work with weights or pulleys was allowed until postoperative six weeks. Active FF and passive external rotation were allowed at six weeks postoperatively, and active movement in all directions was allowed at three months postoperatively. Contact sports or “at risk” work were not allowed for six months.

### Clinical outcome and imaging evaluation

Preoperative and postoperative clinical results were assessed using a visual analog scale (VAS) for pain and instability. Active and passive shoulder motion, including forwarding flexion (FF), abduction(AB), external rotation at the side (ERs), external and internal rotation at 90° of abduction (ERa and IRa, respectively) were assessed preoperatively and at the final follow-up. Quantitative muscle strength of the rotator cuff was assessed using a Biodex System (Biodex Medical Inc., New York, USA). Elevation strength was tested with the patient in a seated position, with the arm flexed to 90° in the scapular plane. External and internal rotation were tested with the shoulder in a neutral position and the elbow in 90° of flexion. Complications were recorded. Also the Rowe scale, American shoulder and elbow surgeons (ASES), Walch-Duplay score were used for clinical assessment.

CT scans were offered for all the patients at postoperative 1 day, 6 months, one year and two years. The ideal position was defined as below the glenoid equator in the axial plane [17] and flush to the glenoid rim in the horizontal plane [18]. Grafts were judged to be lateral or not lateral to the humeral head circumference. Graft positioning was classified into three categories: flush and too media/lateral if it lay 3 mm or more medial/lateral to the rim. Graft healing was assessed by the same imaging studies performed at two years postoperatively according to Hovelius et al. [19]. Humeral head degeneration was assessed according to the standard of Samilson and Prieto et al. [20].

### Statistical analysis

A statistical analysis was performed to investigate the relationship between variables. A paired t-test was performed to assess the differences between pre- and Postoperative VAS score, ROM, strength and functional scores measurements and shoulder scores. It was also used for analysis of imaging measurements. Significance was set at *P < 0.05*.

## Results

### Demographic data

Fifty cases were included in this study. Among them, 39 cases were males, and 11 cases were females with an average age of 24.8 ± 4.8 years (ranged from 18 to 36 years). A total of 45 cases involved the left side, and five cases the right side. The glenoid deficit area measured from the pre-operative CT scans by a bare area method on the en-face view of the 3D CT ranged from 15 to 32%, with an average of 24.3% ± 3.8%. The recorded times of dislocation were from 18 to 25, with an average of 18.2 ± 4.1. 31 cases had their Beighton score over 4. All the cases had their ISIS score over 6(Table 1). The mean follow-up period was 25.0 ± 6.5 months (range, 18–30 months).

**Table 1:** Demographic Data

	All patients(*n* = 50)
Patient characteristics	
Age, y	24.8 ± 4.8 (18–36)
Sex(male/female), n	39/11
Laterality (right/left), n	5/45
Glenoid deficit area, %	24.3 ± 3.8(15–32)
Recorded times of dislocation, n	20.2 ± 4.1(18–25)
Beighton Score ≥ 4, %	62
ISIS score ≥ 6, %	100
Follow-up, m	15.0 ± 6.5(6–32)

Data are reported as mean with the range in parentheses unless otherwise indicated

### Subjective pain

VAS scores for pain during motion decreased from a mean of 2.8 (range, 0–6) preoperatively to 1.5 (range, 0–2) at the final follow-up (*P<0.05*). The improvement in pain during motion was statistically significant (Table 2).

**Table 2:** Clinical outcomes for patients underwent modified suture button arthroscopic Latarjet procedure

Variable	Preoperative	Postperative	*P* Value
VAS during motion	2.8 ± 1.8	1.5 ± 1.1	<.001^*b*^
ROM,deg.(°)			
FF	175 ± 17	172 ± 15	0.325
AB	125 ± 15	129 ± 17	0.215
ERs	57 ± 14	45 ± 11	<.001^*b*^
ERa	78 ± 12	63 ± 16	<.001^*b*^
IRa	65 ± 11	68 ± 13	0.355
ASES score	80.2 ± 16.2	95.2 ± 5.6	<0.001^*b*^
Rowe score	40.2 ± 9.8	94.5 ± 2.7	<0.001^*b*^
Walch-Duplay score	67.5 ± 10.2	95.6 ± 3.2	<0.001^*b*^
Complications, %	–	4	–
Stiffness(n)	–	2	–

All data are presented as mean ± SD. *AB* abduction, *ER* external rotation, *FF* forward flexion, *IR* internal rotation, *ERa* external rotation in abduction, *ERs* external rotation at the side, *IRa* internal rotation in abduction

^b^Statistically significant (*P<.05*)

### Range of motion

The mean preoperative active FF, AB, ERs, ERa, IRa were 175 ± 17°, 125 ± 15°, 57 ± 14°, 78 ± 12°, 65 ± 11°respectively. The mean postoperative FF, ERs, ERa, IRa were 172 ± 15°, 129 ± 17°, 45 ± 11°, 63 ± 16°, 68 ± 13° respectively. There was a significant restriction of the affected shoulder active mobility in ERs, ERa postoperatively (*P<0.05*), the other values were not significantly different pre-and postoperatively (*P>0.05*) (Table 2).

### Overall scores

At the final follow-up, the ASES score, Rowe score, the Walch-Duplay score increased significantly from 80.2 ± 16.2, 40.2 ± 9.8, 67.5 ± 10.2 to 95.2 ± 5.6, 94.5 ± 2.7, 95.6 ± 3.2 respectively (*P<0.05*) (Table 2).

### Complications

No postoperative infection, axillary nerve injury, and bone non-union were observed. Stiffness was found in two cases(one case <120°FF and one case<90°AB), they got greatly improved by physical therapy. The overall complication rate was only 4%(2 of 50 cases) (Table 2).

### Radiologic assessment

CT scans in the sagittal view at postoperative day 1 showed grafts of 44 cases (88%) were positioned between the level of 2:30 and 4:20 o’clock (Fig. 3), which is the ideal position according to Casabianca et al. [21] . Two cases (4%) were positioned above this level and four (8%) below (Table 3).

**Table 3:** Coracoid bone graft position in relation to the glenoid evaluated on postoperative CT scans performed postoperative 1 day

Coracoid bone graft positioning	No. of shoulders (*N* = 50)	%
Sagittal plane		
Between the level of 2:30 and 4:20 o’clock	44	88
Above the level of 2:30	2	4
Below the level of 4:20	4	8
Axial plane		
Flush to the glenoid surface	40	80
Too medial (> 3 mm medial to the glenoid rim)	0	0
Too lateral (> 3 mm lateral to the glenoid rim)	10	20

**Fig. 5:**
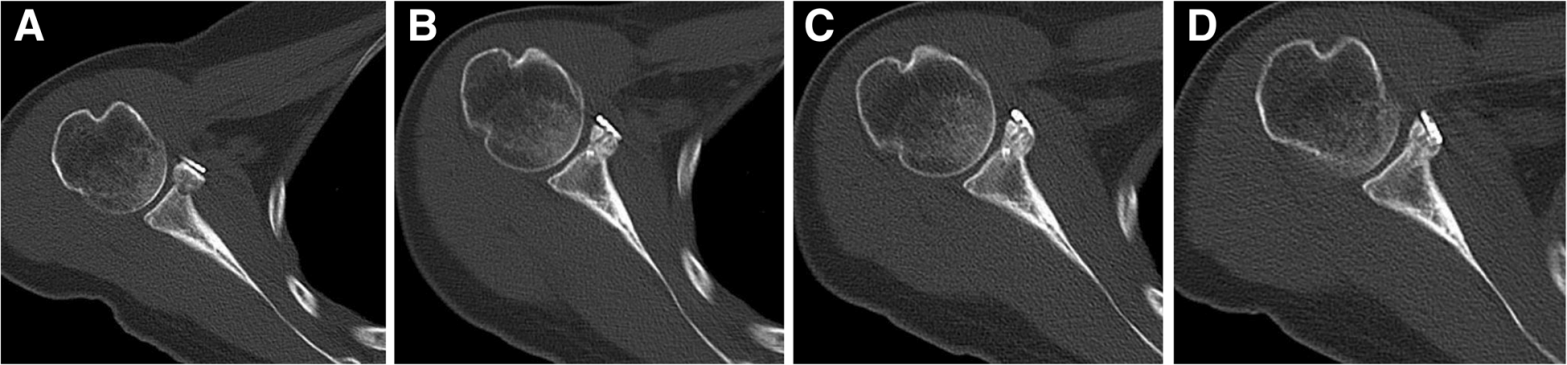
The remodeling process of grafts positioning too laterally in one case. **a** In the axial view at postoperative day 1, the graft was placed obviously higher than the level of glenoid rim. **b** The graft became remodeled and the distance higher than the level of the glenoid rim decreased significantly at postoperative six months. **c** The graft became further remodeled and nearly being flushed with the glenoid rim at postoperative one year. **d** The graft was flush with the glenoid rim at postoperative two years without causing any glenohumeral degenerative changes

**Fig. 6:**
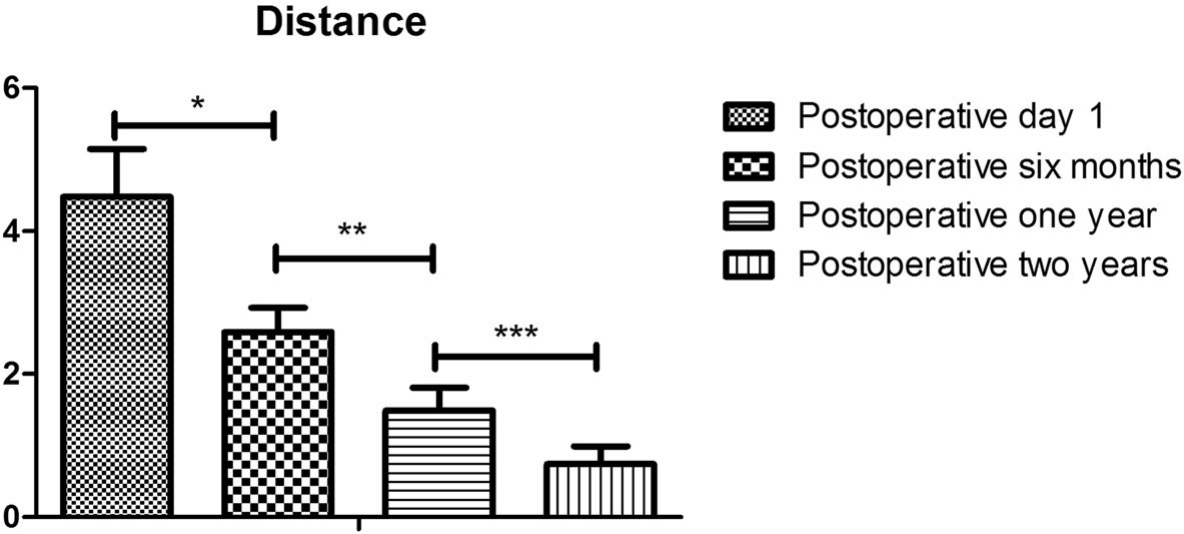
Evolution of the average distances of the grafts positioning too laterally in the axial plane at the postoperative day 1, six months, one and two years assessed from CT scans

In the axial view at postoperative day 1, grafts in 40 cases (80%) were flush with the glenoid rim, 10 cases (20%) were considered as too lateral, no one case as too medial (Table 3). The ten grafts positioning too laterally were placed higher than the level of glenoid rim with an average of 4.48 ± 0.67 mm (Table 4). However, these grafts became remodeled and the distance higher than the level of glenoid rim decreased to 2.59 ± 0.34 mm, 1.49 ± 0.32 mm and 0.74 ± 0.25 mm at the follow-up of postoperative six months, one year, two years respectively (Figs. 5 and 6) (Table 4). Finally, grafts were flush with the glenoid rim.

**Table 4:** Evolution of the distances of the grafts positioning too laterally in the axial plane at the postoperative day 1, 3 months, 6 months and 1 year assessed from CT scans

Time	Distance(mm)	P
Postoperative day 1	4.48 ± 0.67	*–*
Postoperative 6 months	2.59 ± 0.34	*P*^*a*^<*0.021*
Postoperative 1 year	1.49 ± 0.32	*P*^*b*^<*0.001*
Postoperative 2 years	0.74 ± 0.25	*P*^*c*^<*0.001*

^a^, ^*b*^, and ^*c*^ mean the distance between the postoperative day 1 and Postoperative 6 months, the distance between the postoperative 6months and Postoperative 1 year and the distance between the postoperative 1 year and Postoperative 2 years are significantly different (*P < 0.05*)

No degeneration was observed in all the cases. Bone union was achieved in all the fifty cases.

## Discussion

Traditionally, two metallic screws are used to fix the bone graft in an arthroscopic Latarjet procedure. This fixation is reliable; however, some complications, such as irritation to the supraspinatus tendon or humeral head, and cartilage injury of the humeral head, may occur [22, 23]. Some scholars used bioabsorbable screws for fixation instead of metallic screws. Such fixation method can avoid the noted complications efficiently and delay humeral head degeneration [11]. However, these fixation methods provide rigid fixing; if the initial position is achieved, the position cannot be adjusted unless a second surgery is performed [23]. Moreover, the position of the grafts is of great importance for the long-term efficacy of the arthroscopic Latarjet procedure. If the position is not good, it will result in the failure of the operation and several complications. Hovelius et al. and Allain et al. [19, 24] have reported a failure rate of 58 and 36% in the final fixed position, respectively. On the axial plane, if the position is too lateral, it will cause impingement and restrict the rotation of the humeral head. Meanwhile, if the position is too medial, shoulder dislocation will not be effectively prevented. On the en-face view, the optimal position is said to be between the level of 2:30 and 4:20 o’clock according to Casabianca et al. [21], and the grafts positioned in this range will provide better shielding effect.

As noted above, the fixation method and position are two important factors. How to deal with them is difficult and undoubtedly important. Regarding the fixation method, Boileau et al. [14] presented a suture button fixation method using titanium buttons in arthroscopic Latarjet procedure, which seems novel and useful in preventing complications. However, this method needs six to seven portals and some special instruments are needed. We modified this method using only three portals and fixed the bone grafts through special procedures, in which an anti-rotation design was added. In our point of view, the suture button technique provided minimal motion of bone grafts, which allowed small displacement if grafts were fixed laterally or medially so that complications due to rigid fixation can be avoided. Meanwhile, total freshening was made for the bone graft intra-operatively by open technique, which can save a lot of time and allow excellent healing and remodeling of the interface between the glenoid and grafts and finally became flush with the glenoid. In addition, several high-strength sutures communicated the glenoid with the bone graft, which guaranteed the interface to bear a big shear force, so that solid fixation can be confirmed. All cases in our study achieved bone union without fibrous union and non-union due to the above reasons. In Boileau’s study, no bone nonunion was observed and the fibrous union rate was 9% [14]. Maybe the difference between ours and his is because some steps different during the procedure including graft obtaining, fixation suture numbers and suture anchors usage. Moreover, the bone tunnels in the graft and glenoid were prepared in advance during the operation to avoid deviation of position fixing in the sagittal view.

Cases in which the bone grafts were fixed more upwardly or laterally were observed in our study. Some factors can be accounted for this phenomenon. Arthroscopic Latarjet technique is relatively complicated with a long learning curve. During the operation, the grafts are prone to be displaced to the glenoid rim due to visual difference; these grafts are believed to be impinged with the humeral head, thus causing cartilage damage and secondary joint degeneration according to Spoor et al. [22]. The grafts positioned laterally became flush with the glenoid rim. The phenomenon was due to the possible reasons listed below: 1) The displaced distance was not very large, 2) Even if the coracoid graft had been fixed laterally, debridement would also be performed until the grafts reached the glenoid cartilage level, 3) Our technique provided minimal motion of bone grafts, which allowed small displacement if they were fixed laterally or medially, 4)As the grafts became gradually absorbed and healed, the parts located above the glenoid level became remodeled. To our knowledge, this is the first case series presenting such phenomenon which could provide a new concept for the Latarjet procedure.

Despite the merits shown in our study, it still has some limitations. Firstly, no comparison was made between our technique and other techniques even open technique. Second, the follow-up is relatively short, and observation of long-term efficacy is still needed. Furthermore, this study was retrospectively carried out. More prospective research including randomized controlled trials should be performed to provide more evidence.

## Conclusions

The MSB arthroscopic Latarjet procedure provides excellent outcome with few complications, and no degenerative changes were observed in the follow-up. Moreover, the transplanted graft fixed too laterally presented a phenomenon of remodeling and became more flushed with the glenoid rim over time.

## Additional files

Additional file 1: Surgery Demo A short video for how to do the MSB Surgery. (MP4 16 mb)

Additional file 2: Surgery Schematic A video shows the graph of steps for the MSB surgery. (MP4 1 mb)

## Abbreviations

Era: External rotation in abduction
Ers: External rotation at the side
FF: Forward Flexion
MSB: Modified suture button
